# The accuracy of microRNA-210 in diagnosing lung cancer: a systematic review and meta-analysis

**DOI:** 10.18632/oncotarget.11446

**Published:** 2016-08-20

**Authors:** Huqin Yang, Huijuan Wang, Chao Zhang, Zhaohui Tong

**Affiliations:** ^1^ Department of Respiratory and Critical Care Medicine, Beijing Institute of Respiratory Medicine, Beijing Chao-Yang Hospital, Capital Medical University, Beijing, 100020, China

**Keywords:** microRNA-210, lung cancer, diagnosis, meta-analysis, systematic review

## Abstract

Studies examining the diagnostic value of microRNA-210 for lung cancer have yielded inconsistent results. Here, we performed a meta-analysis to assess the diagnostic accuracy of microRNA-210 for lung cancer. Nine eligible studies involving 993 patients (554 lung cancer patients and 439 non-cancer patients) were independently identified, and the quality of these studies was assessed according to Quality Assessment of Diagnostic Accuracy Studies (QUADAS-2) guidelines. The pooled sensitivity, specificity, positive likelihood ratio, negative likelihood ratio, and diagnostic odds ratio were 0.66 (95% CI, 0.57 to 0.75), 0.82 (95% CI, 0.72 to 0.89), 3.64 (95% CI, 2.54 to 5.21), 0.41 (95% CI, 0.34 to 0.51) and 8.78 (95% CI, 6.10 to 12.66), respectively. The area under the summary receiver operator characteristic curve was 0.80 (95% CI, 0.76 to 0.83). These results indicated that microRNA-210 had moderate diagnostic value for lung cancer. Additional prospective studies are needed to confirm the diagnostic value of microRNA-210.

## INTRODUCTION

Lung cancer is the leading cause of life-threatening malignant diseases in humans. The American Cancer Society estimates that lung cancer incidences in 2012 were 125 per 100,000 men (0.125%) and 50 per 100,000 women (0.05%) [1]. Lung cancer accounted for 28% of cancer-related deaths in 2015, which can be partly attributed to the lack of effective molecular markers for early tumor diagnosis. As a result, lung cancer diagnosis is often delayed in patients who are initially asymptomatic or show non-specific symptoms. Fortunately, as the prevalence of lung computed tomographic (CT) screening in smoking adults has increased, rates of early diagnosis in lung cancer have also increased [2]; additionally, 5-year survival rates increased from 12% in 1975–1977 to 18% in 2005–2011 [1]. However, CT scans have many shortcomings, including high cost, radiation exposure, and low specificity [3].

MicroRNAs (miRNAs) are endogenous non-coding small RNAs ranging from 19 to 25 nucleotides in length [4]. Approximately 50% of miRNAs are located in tumor-related regions [5]. Circulating miRNAs in serum or plasma and miRNAs identified in sputum samples remain stable even under harsh experimental conditions [6–8]. Many studies have indicated that miRNA levels in fluid samples might be promising biomarkers for the detection of lung cancer because measurements of miRNA expression, which varies widely, are noninvasive, quantitative, and reproducible [9–11].

Xing *et al*. first demonstrated that the miRNA miR-210 distinguished lung squamous cell carcinoma patients from normal controls with 58% sensitivity and 79% specificity [12]. Since then, many additional studies have suggested that miR-210 might serve as a biomarker for lung cancer [12–20]. Furthermore, Osugi *et al*. found that high miR-210 expression was correlated with increased lymph node metastasis, later cancer stages, and poor prognosis in patients with non-small-cell lung cancer (NSCLC), suggesting that miR-210 might be a prognostic biomarker in these patients [21].

However, inconsistencies exist among studies that have examined the diagnostic value of miR-210. Here, we performed, to our knowledge, the first meta-analysis of all eligible studies to assess the diagnostic accuracy of miR- 210 for lung cancer.

## RESULTS

### Data extraction

**Figure 1:**
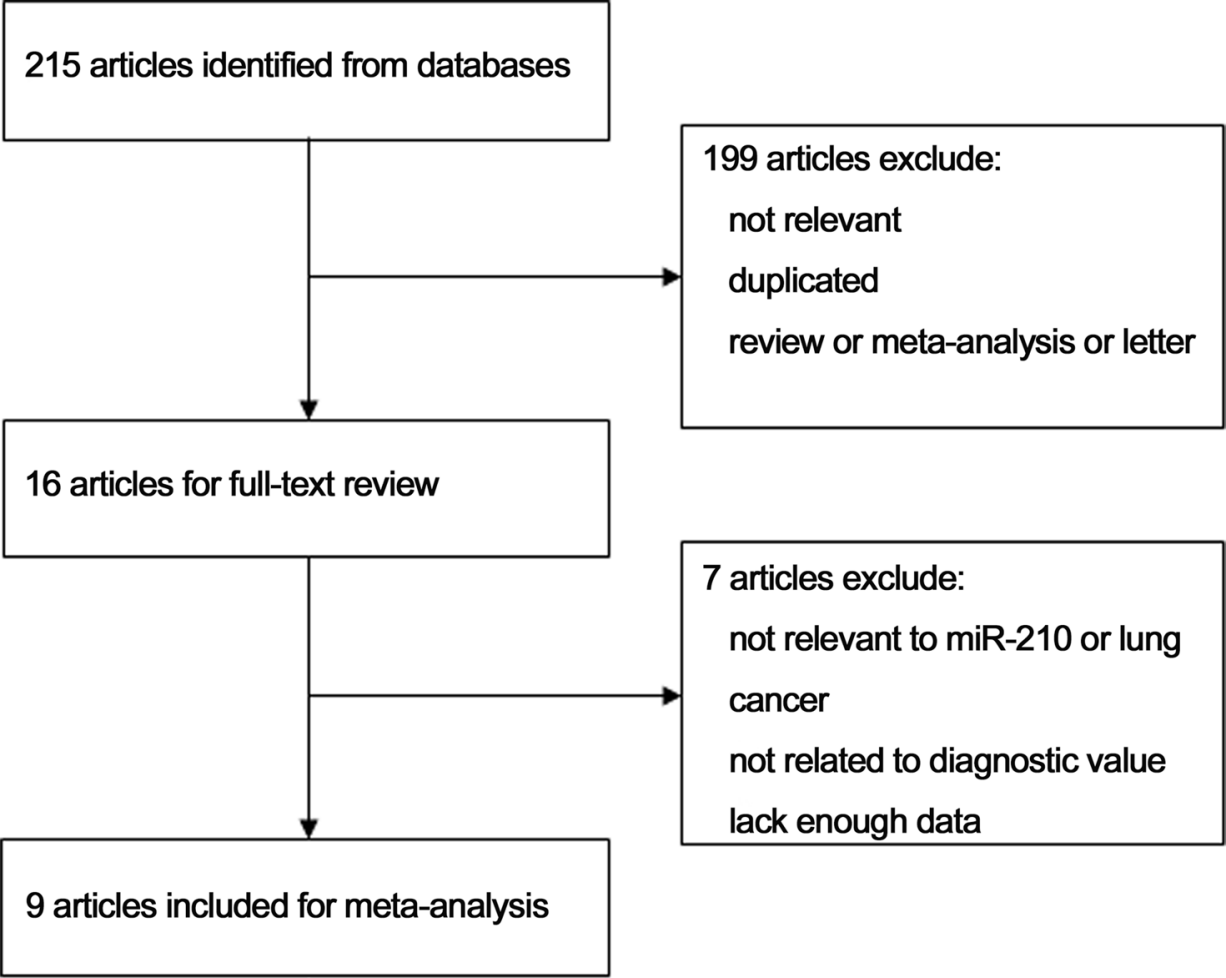
Flow chart of the study selection process

215 potentially relevant articles were identified using several databases. As shown in Figure 1, 16 articles were retrieved and read in detail. Among these 16 papers, four that had no diagnostic indicators and three that lacked 2 × 2 contingency tables were excluded. Ultimately, the nine remaining articles were included in the meta-analysis.

### Data characteristics and quality assessment

**Figure 2:**
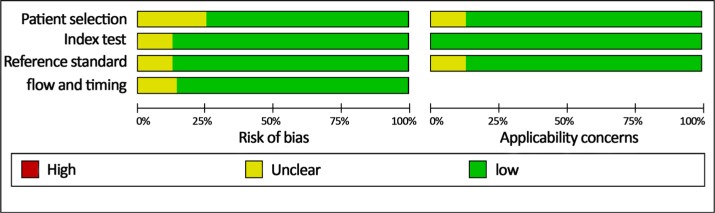
Quality of selected studies according to QUADAS-2 guidelines

The nine studies, sorted by publication year (ranging from 2010 to 2016), are shown in Table 1. A total of 993 patients were involved in these studies, including 554 lung cancer patients and 439 non-cancer patients. Two of the studies were conducted in China and seven in the United States. Two studies investigated both NSCLC and small-cell lung carcinoma (SCLC), while seven examined only NSCLC. Four papers included stage I–II lung cancer patients, and five included stage I–IV patients. The number of patients in each study ranged from 75 to 156, and the median number of samples per study was 96. Five studies examined sputum samples and four examined blood samples. Reverse transcription-quantitative polymerase chain reaction (RT-qPCR) was used to measure miR- 210 expression in all nine studies; eight of them used the TaqMan kit and one used the SYBR-Green assay. Endogenous reference genes also differed among the studies, with five using U6 and four using miR-16. Quality Assessment of Diagnostic Accuracy Studies (QUADAS-2) guidelines indicated that the selected studies were of high quality (Figure 2).

**Table 1:** Summary of included studies using miR-210 as a biomarker of lung cancer

Fisrt author	Year	Country	Cancer type	Stage	Patients (controls)	Mean age (years)	Male	Specimen	Normalizar	Detection method	TP	FP	FN	TN	Sensitivity(%)	Specificity(%)
Zhu W	2016	China	NSCLC	I–IV	112 (40)	58.5	53.6%	serum	U6	QT-PCR (TaqMan)	38	0	74	40	33.9%	100.0%
Xing L	2015	USA	NSCLC	I–II	60 (62)	67.3	63.3%	sputum	miR-16	QT-PCR (TaqMan)	45	9	15	53	75.3%	85.9%
Shen J	2014	USA	lung cancer	I–IV	66 (68)	64	56.1%	sputum	U6	QT-PCR (TaqMan)	43	18	23	50	65.8%	73.8%
Li N	2014	USA	lung cancer	I	35 (40)	68.9	62.9%	sputum	U6	QT-PCR (TaqMan)	20	4	15	36	57.1%	90.0%
Li ZH	2013	China	NSCLC	I–IV	60 (30)	53.9	70.0%	serum	miR-16	QT-PCR (SYBR Green)	47	8	13	22	78.7%	74.0%
Anjuman N	2013	USA	NSCLC	I	39 (42)	65.6	59.0%	sputum	U6	QT-PCR (TaqMan)	27	10	12	32	69.2%	76.2%
Shen J	2011	USA	NSCLC	I–IV	76 (80)	67.9	55.3%	plsma	miR-16	QT-PCR (TaqMan)	56	44	20	36	74.1%	69.0%
Shen J	2011	USA	NSCLC	I–IV	58 (29)	67.8	65.5%	plsma	miR-16	QT-PCR (TaqMan)	46	7	12	22	79.3%	75.9%
Xing L	2010	USA	NSCLC	I	48 (48)	NA	NA	sputum	U6	QT-PCR (TaqMan)	28	10	20	38	58.3%	79.2%

### Diagnostic accuracy of microRNA-210 in lung cancer

**Figure 3:**
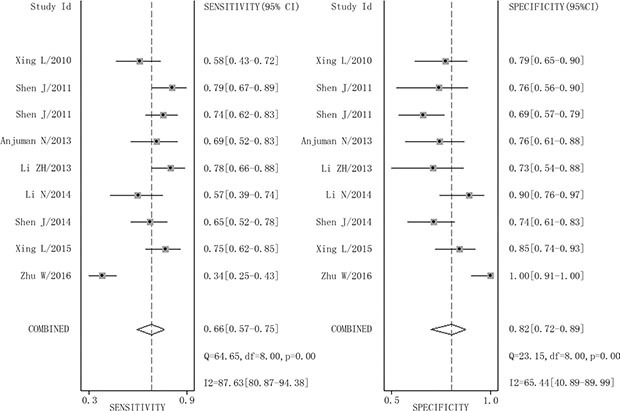
Forest plots of sensitivity and specificity for miR-210 in the diagnosis of lung cancer

**Figure 4:**
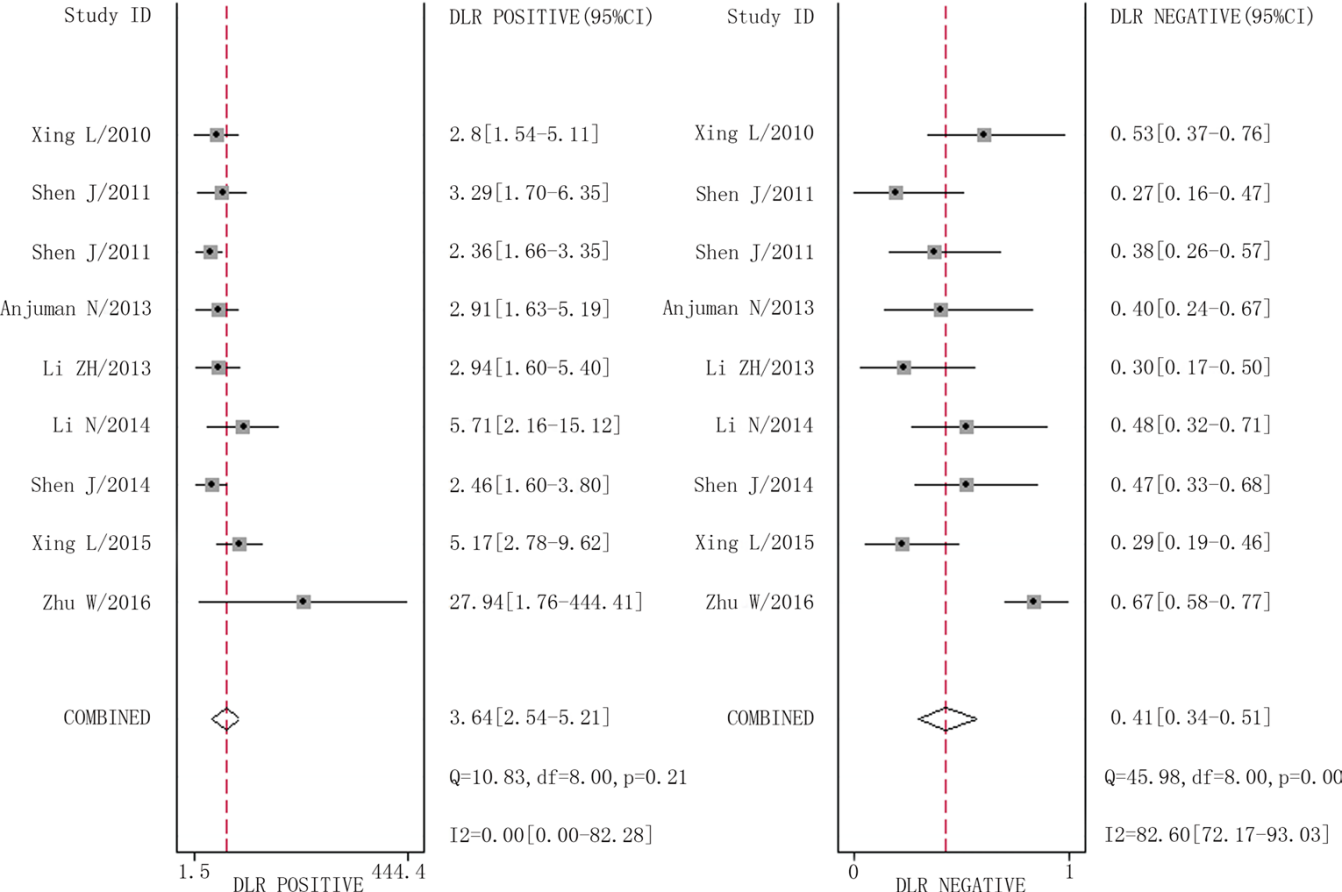
Forest plots of positive likelihood ratio and negative likelihood ratio for miR-210 in the diagnosis of lung cancer

**Figure 5:**
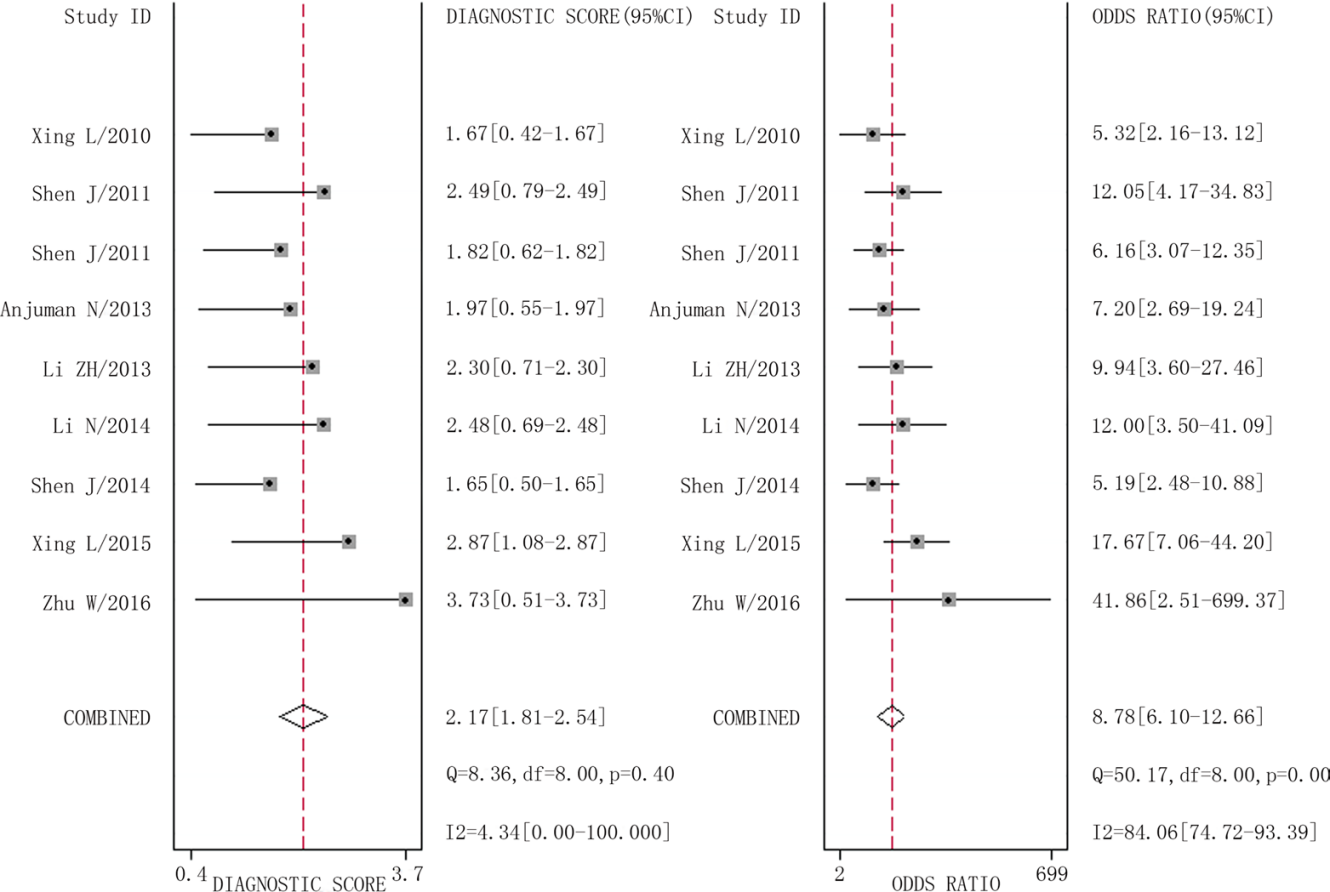
Forest plots of diagnostic odds ratio for miR-210 in the diagnosis of lung cancer

**Figure 6:**
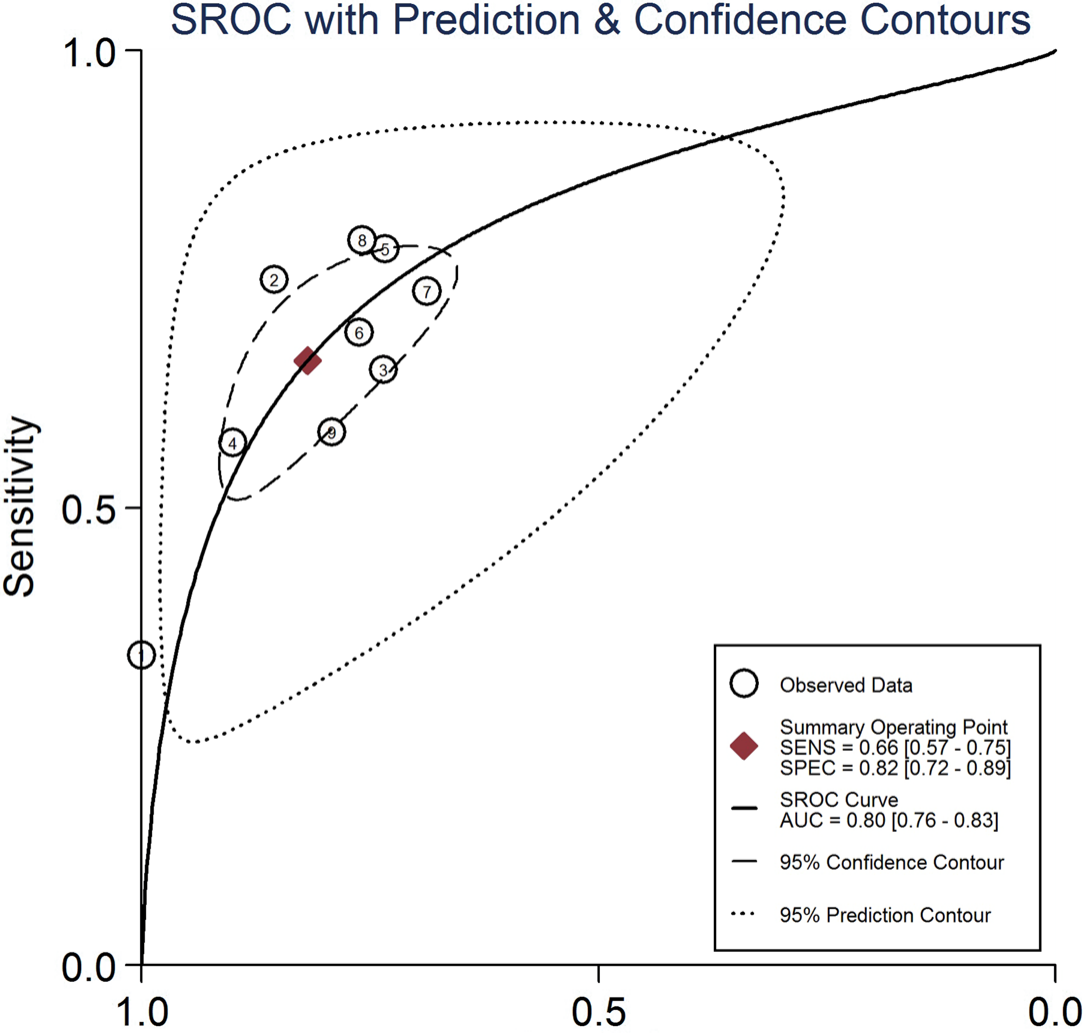
Summary receiver operating characteristic curve for miR-210 in the diagnosis of lung cancer

**Figure 7:**
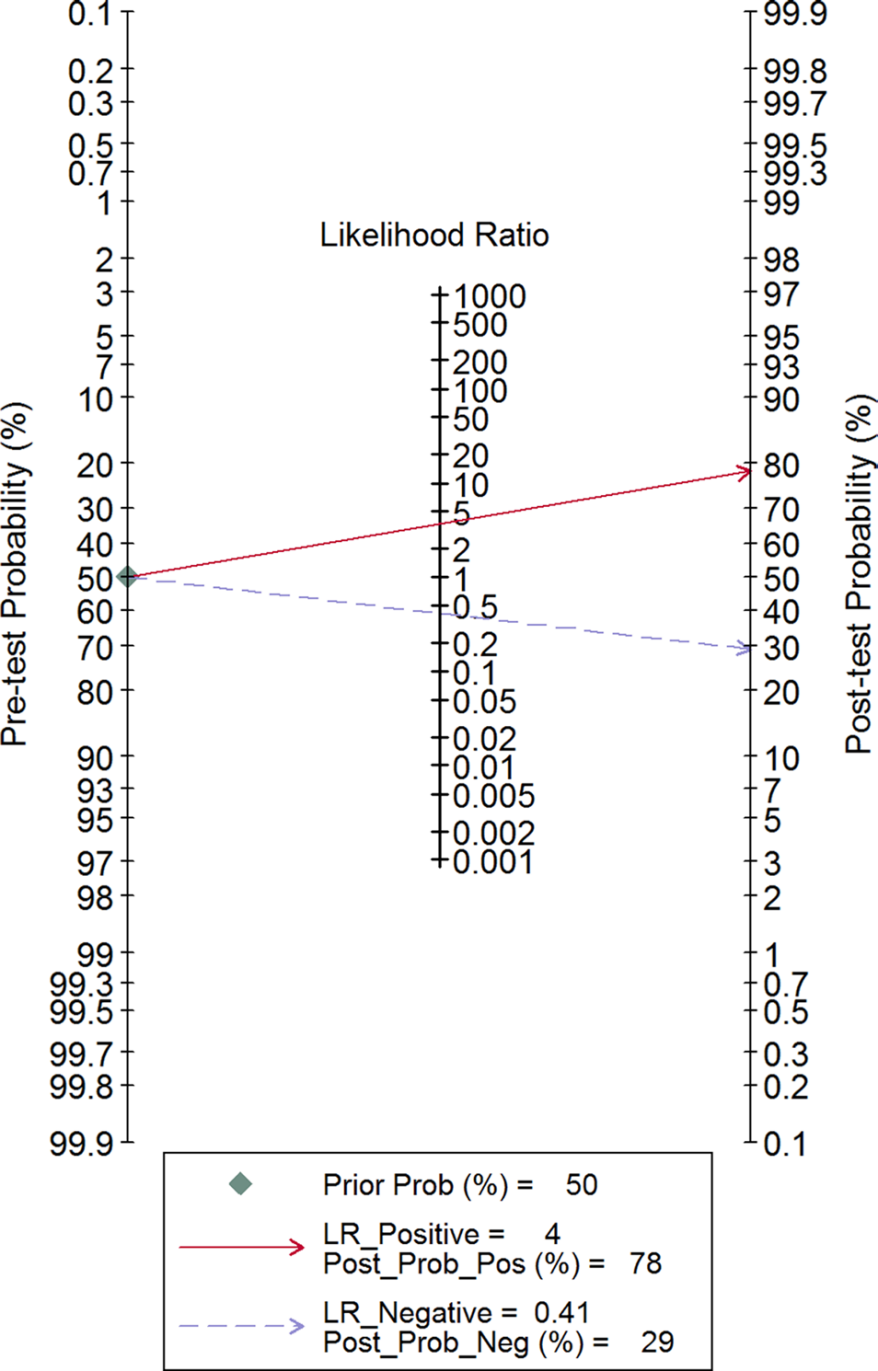
Fagan's nomogram for likelihood ratios

**Figure 8:**
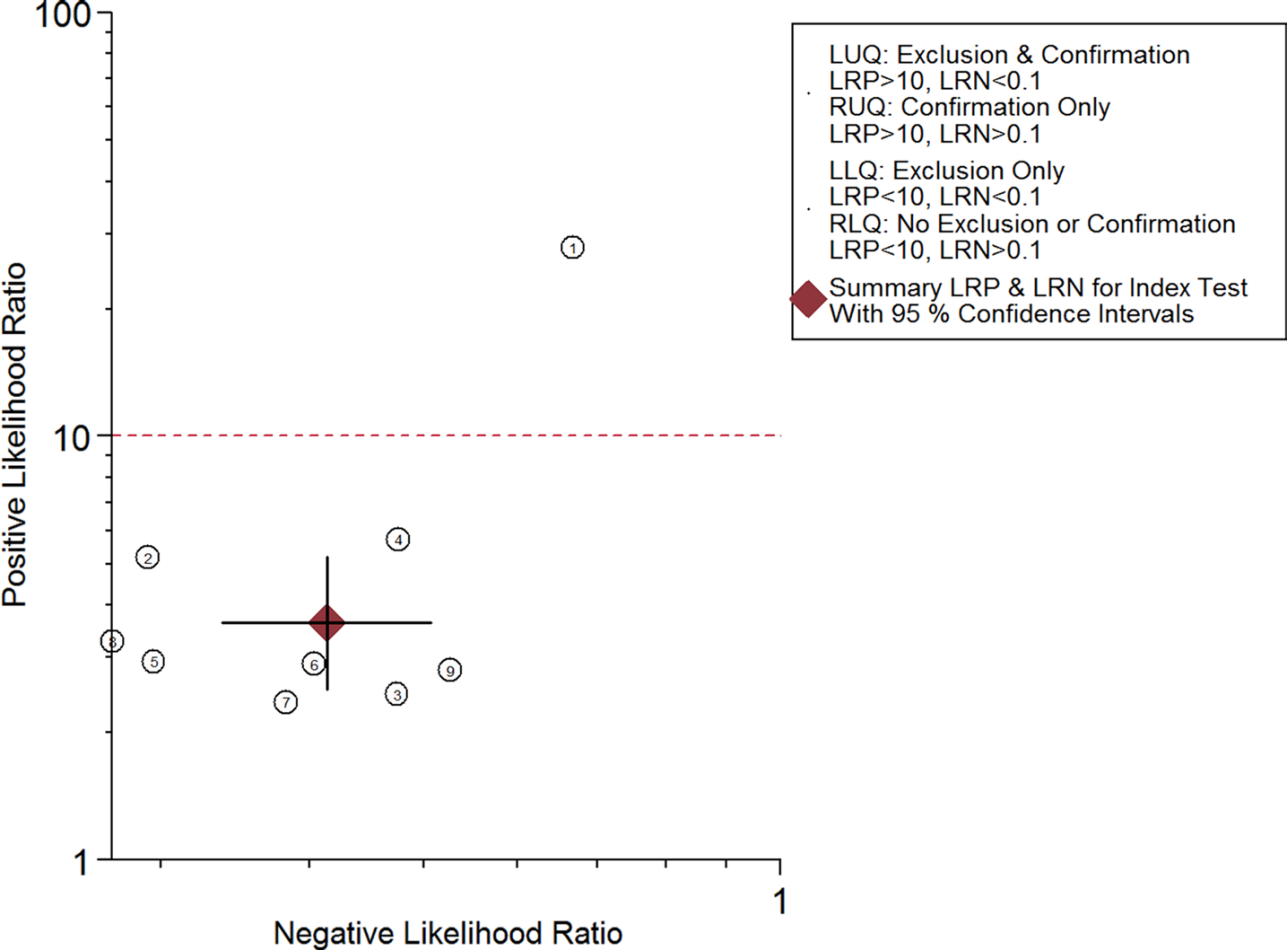
Likelihood ratio scattergram

**Figure 9:**
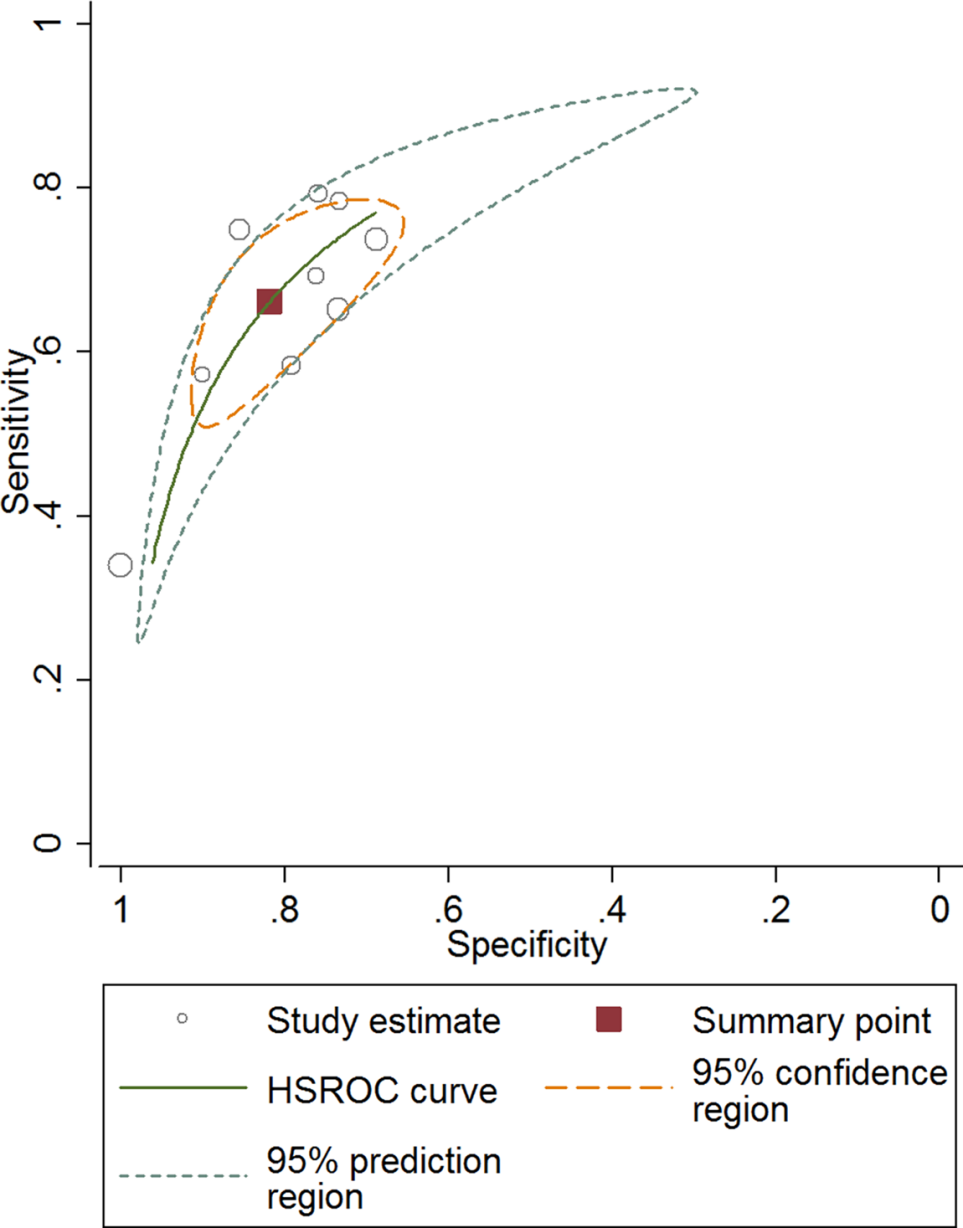
Hierarchical summary receiver operating characteristics curve for miR-210 in the diagnosis of lung cancer

The combined pooled sensitivity and specificity for all of the studies were 0.66 (95% confidence interval (CI), 0.57 to 0.75) and 0.82 (95% CI, 0.72 to 0.89), respectively (Figure 3). I^2^ values for sensitivity and specificity were 87.63% (95% CI, 80.87 to 94.38) and 65.44% (95% CI, 40.89 to 89.99), respectively, suggesting significant heterogeneity in sensitivity and specificity. We therefore investigated the source of the heterogeneity. The positive likelihood ratio (PLR) and negative likelihood ratio (NLR) were 3.64 (95% CI, 2.54 to 5.21) and 0.41 (95% CI, 0.34 to 0.51), respectively (Figure 4). The diagnostic odds ratio (DOR) was 8.78 (95% CI, 6.10 to 12.66) (Figure 5). The area under the summary receiver operator characteristic (SROC) curve was 0.80 (95% CI, 0.76 to 0.83) (Figure 6). Figure 7 shows Fagan's nomogram for likelihood ratios, which was used to determine *post*-test probabilities resulting from different pre-test probabilities. A likelihood ratio scattergram was used to determine the clinical values of different diagnostic methods and was divided into four quadrants (Figure 8). The left upper quadrant (LRP > 10, LRN < 0.1) indicates confirmation and exclusion diagnostic value, the right upper quadrant (LRP > 10, LRN > 0.1) confirmation diagnostic value, the left lower quadrant (LRP < 10, LRN < 0.1) exclusion diagnostic value, and the right lower quadrant (LRP < 10, LRN > 0.1) neither confirmation nor exclusion diagnostic value [22]. Eight of the nine studies were plotted in the right lower quadrant, and the remaining study was in the right upper quadrant, indicating that miR-210 had a moderate diagnostic value in lung cancer. As shown in Figure 9, a hierarchical summary receiver operating characteristics (HSROC) curve was constructed. The estimated value of β was 0.26 (95% CI, -0.45 to 0.99), the value of z was 0.73, and the *p*-value was 0.47, implying that the SROC curve was symmetric. The value of Lambda was 2.08 (95% CI, 1.76 to 2.40), which also indicated that miR-210 had moderate accuracy in diagnosing lung cancer.

### Meta-regression and subgroup analysis

Potential heterogeneity in sensitivity and specificity was explored with meta-regression analysis and subgroup analysis. Country, tumor stage, cancer type, specimen, and normalizer were used as co-variants in meta-regression. As shown in Table 2, country, tumor stage, cancer type, specimen, and normalizer did not explain the heterogeneity (*p* > 0.05). Subgroup analysis by country revealed that the pooled sensitivity and specificity in the seven studies conducted in USA were 0.69 and 0.78, respectively; for the two studies conducted in China, these values were 0.49 and 0.89, respectively. Subgroup analysis by cancer type indicated that, in the seven studies of NSCLC patients only, the pooled sensitivity was 0.63 and the specificity was 0.80; in the two studies of both NSCLC and SCLC patients, the pooled sensitivity was 0.62 and the specificity was 0.82. The pooled sensitivity and specificity were 0.66 and 0.83 in the four studies of stage I-II patients 0.62 and 0.77 in the five studies of stage I-IV patients, respectively. Finally, pooled sensitivity, specificity, and area under curve (AUC) for studies in which miR-210 was measured in blood were 0.67, 0.86, and 0.81, and were 0.66, 0.81, and 0.75 for studies in which it was measured in sputum, respectively (Table 3).

**Table 2:** Relative diagnostic odds ratio (RDOR) of covariants in the meta-regression analysis

Var	RDOR	95%CI	*P* value
Country	1.39	(0.11,17.81)	0.63
Stage	1.25	(0.03,50.45)	0.82
Type	0.84	(0.03,22,81)	0.84
Specimen	0.79	(0.01,49.35)	0.83
Normalizar	0.36	(0.03,4.30)	0.22

**Table 3:** Summary results of subgroup analysis for miRNA-210 in the diagnosis of lung cancer

Country	Number of studies	Sensitivity (95%CI)	Specificity (95%CI)	PLR (95%CI)	NLR (95%CI)	DOR (95%CI)
Country						
USA	7	0.69(0.64–0.74)	0.78(0.73–0.82)	3.05(2.49–3.73)	0.40(0.34–0.47)	7.72(5.64–10.57)
China	2	0.49(0.42–0.57)	0.89(0.79–0.95)	6.84(0.50–93.50)	0.46(0.17–1.24)	12.90(3.63–45.79)
Type						
NSCLC	7	0.63(0.59–0.68)	0.79(0.74–0.83)	3.07(2.34–4.02)	0.40(0.26–0.59)	8.68(5.96–12.63)
Lung cancer	2	0.62(0.52–0.72)	0.80(0.71–0.87)	3.53(1.49–7.57)	0.47(0.36–0.62)	6.79(3.15–14.63)
Stage						
Early (I–II)	4	0.66(0.59–0.73)	0.83(0.77–0.88)	3.82(2.76–5.30)	0.41(0.33–0.51)	9.30(5.82–14.87)
All (I–IV)	5	0.62(0.57–0.67)	0.77(0.71–0.82)	2.92(2.29–3.73)	0.41(0.25–0.66)	7.97(5.40–11.77)
Specimen						
Blood	4	0.67(0.47–0.82)	0.86(0.54–0.97)	2.87(1.90–4.33)	0.39(0.20–0.75)	8.49(5.17–13.96)
sputum	5	0.66(0.60–0.72)	0.81(0.75–0.85)	3.26(2.37–4.46)	0.44(0.36–0.53)	7.92(4.89–12.85)
normalizar						
U6	5	0.52(0.46–0.58)	0.82(0.77–0.87)	3.09(2.10–4.55)	0.52(0.40–0.68)	7.47(4.86–11.49)
miR-16	4	0.76(0.71–0.81)	0.76(0.69–0.81)	3.10(2.40–4.01)	0.32(0.25–0.40)	9.70(6.40–14.71)

### Publication bias evaluation

**Figure 10:**
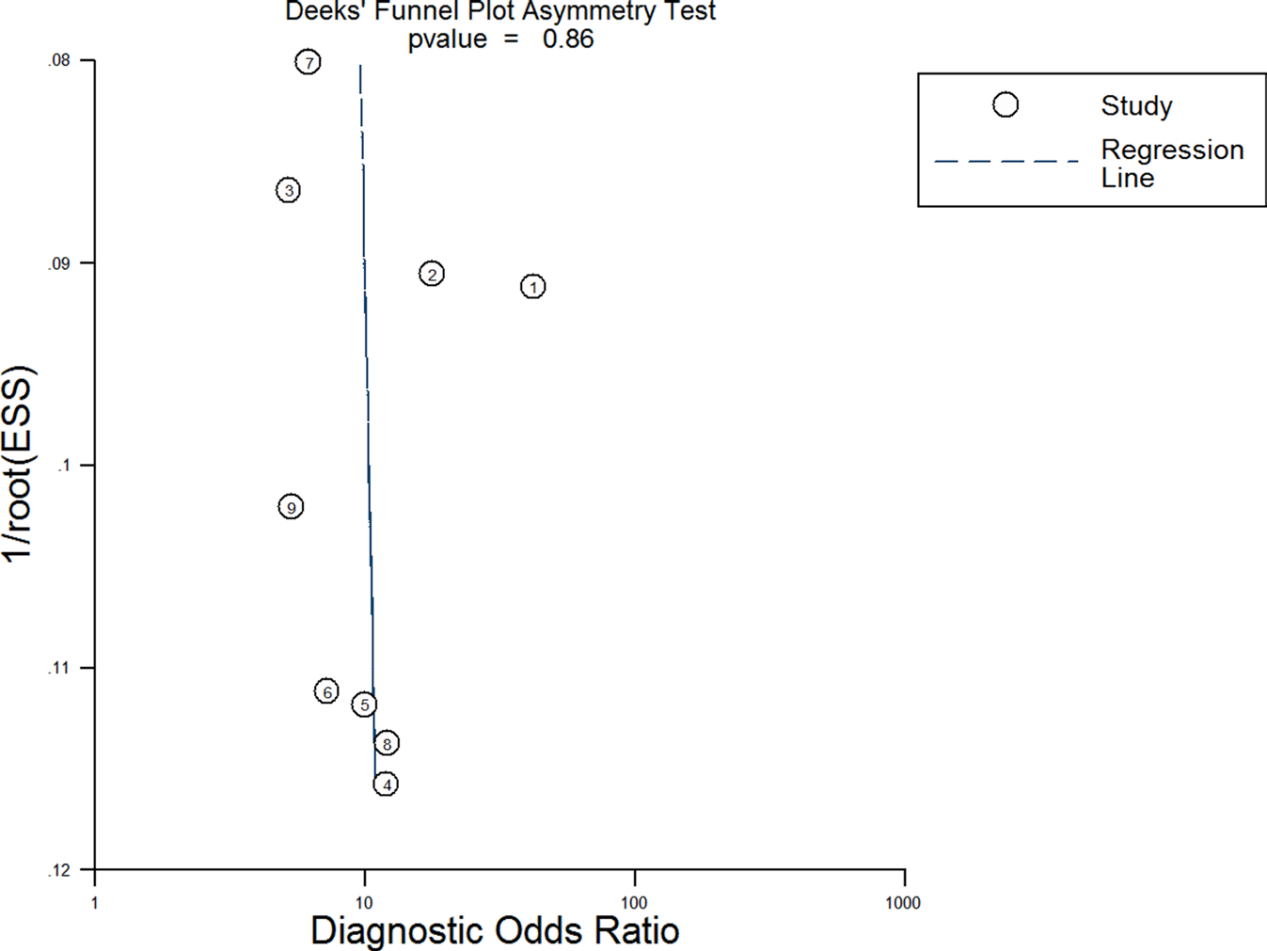
Deeks' funnel plot asymmetry test for assessing publication bias

**Figure 11:**
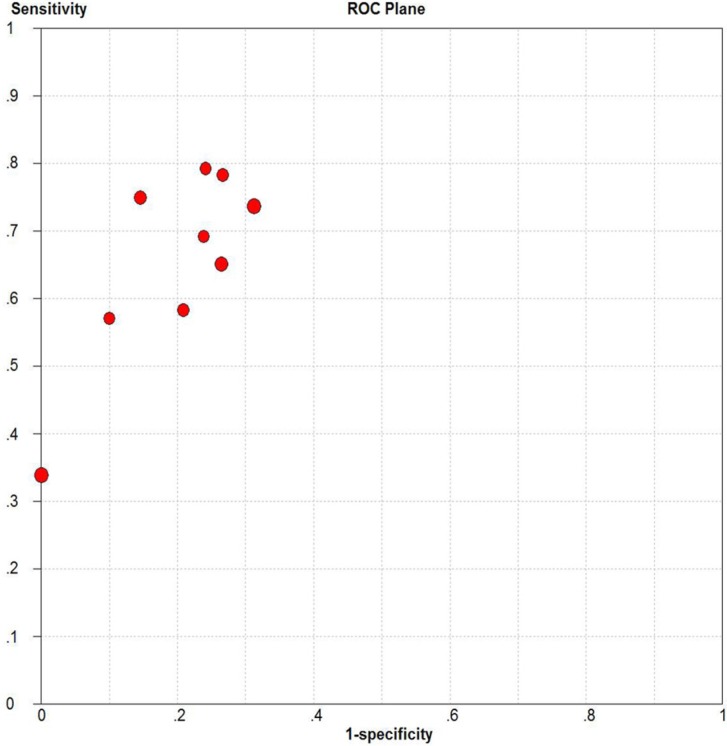
Receiver operating characteristics plane for assessing threshold effects

Deeks' funnel plot asymmetry test was used to assess publication bias (Figure 10). The *p*-value for the linear regression was 0.86, suggesting that there was no publication bias. The ROC plane generated by meta-disc software, which is shown in Figure 11, did not show a “shoulder-arm” shape. A Spearman rank correlation was then performed to test the threshold effect. The Spearman correlation coefficient was 0.633 (*p* = 0.067), suggesting that there was no threshold effect.

## DISCUSSION

Due to the high incidence of lung cancer and low survival rates, lung cancer screening is particularly important [1]. However, affordable, repeatable, and precise detection methods are lacking. Although miRNAs might have particularly high diagnostic values [23–25], the clinical utility of miR-210 expression for diagnosing lung cancer remains controversial [12–20]. This study is the first meta-analysis to evaluate the accuracy of miR-210 in diagnosing lung cancer.

Nine studies conducted between 2010 and 2016 involving a total of 993 patients (554 lung cancer patients and 439 non-cancer patients) were included in this meta-analysis. AUC is widely recognized as a useful index for evaluating the accuracy of diagnostic tests; AUCs between 0.5 and 0.7, 0.7 and 0.9, and greater than 0.9 indicate low, moderate, and high diagnostic value, respectively. Here, we found that the area under the SROC curve was 0.80, suggesting that miR-210 has a moderate diagnostic value for lung cancer. The pooled sensitivity and specificity values for all of the studies combined were 0.66 and 0.82, respectively. As shown in Figure 7, assuming a pre-test probability of 78% and a PLR of 4.0, measuring miR-210 expression to diagnose lung cancer would raise the *post*-test probability to 78%. DOR, a comprehensive evaluation index in diagnostic tests, was used to investigate multiple relationships between chances of obtaining positive and negative results. The pooled DOR was 8.78, indicating that MiR-210 might be a useful diagnostic biomarker for lung cancer. The HSROC model, which was used to further confirm these findings, yielded similar sensitivity (0.66) and specificity (0.82) values. When considered together, these analyses indicate that miR-210 had a moderate accuracy for diagnosing lung cancer.

Although there was significant heterogeneity in sensitivity and specificity in this study, meta-regression did not reveal any factors that accounted for this heterogeneity. We then performed subgroup analyses by country, tumor stage, cancer type, specimen, and normalizer. Although specimen type did not contribute to heterogeneity in meta-regression analysis, in subgroup analysis, blood-based tests yielded a sensitivity and specificity of 0.67 and 0.86, respectively, while sensitivity and specificity of sputum-based tests were 0.66 and 0.81, respectively. This suggests that the diagnostic value of miRNA-210 in blood samples was slightly higher than in sputum samples, which is consistent with previous results [26]. In addition, the diagnostic value of miR-210 might differ depending on cancer stage. In early stages (I–II), the sensitivity, specificity, and DOR were 0.66, 0.83, 9.30, but when stages III-IV were included, the sensitivity, specificity and DOR were 0.62, 0.77, 7.97, respectively (Table 3), suggesting that miR-210 expression might assist in lung cancer diagnosis particularly in early-stage patients. In contrast, previous studies found that miR-210 was a poor prognostic biomarker for lung cancer and was more highly expressed in later stages [21, 27]. However, another study suggested that miR-210 had a positive prognostic impact in lung cancer [28]. Additionally, miR-210 may act as both an oncogene and a tumor suppressor by affecting hypoxia, which in turn influences both cell death and survival [29]. More studies are needed to confirm the prognostic value of miR-210 and its mechanism of action in lung cancer.

Although the diagnostic value of individual miRNAs in lung cancer is limited, many studies have shown that panels of miRNAs or other tumor biomarkers improve diagnostic efficiency. For instance, Xing *et al*. found that a combination of three miRNAs (sputum miR-21, 31, and 210) yielded an AUC of 0.92, 82.93% sensitivity, and 87.84% specificity, while an miR-210 assay alone resulted in an AUC of 0.85, 75.27% sensitivity, and 85.88% specificity. Similarly, Zhu *et al*. found that examining a combination of four miRNAs (serum miR-182, miR-183, miR-210, and miR-126) with carcinoembryonic antigen (CEA) levels increased diagnostic value, with an AUC of 0.975, 88.5% sensitivity, and 92.5% specificity. In contrast, the AUC was 0.65, sensitivity was 33.9%, and specificity was 100.0% for miR-210 alone. Thus, examining several biomarkers in a single test might increase the efficiency of lung cancer diagnoses.

The mechanism by which miR-210 up-regulation occurs in lung cancer is not fully understood. Hypoxia might partially explain the association between increased miR-210 expression and lung cancer [29]. The expression of some miRNAs, called hypoxia-regulated miRNAs (HRMs), is related to hypoxia, which is an independent prognostic factor for various tumors. Hypoxia-inducible factor-1 alpha (HIF-1α) and HIF-2α induce the expression of miR- 210, which is an HRM, in both normal and low oxygen-transformed cells [30–31]. miR-210 expression also affects cell cycle progression, cell survival, differentiation, DNA repair, and angiogenesis [29]. The mechanisms through which miR-210 affects tumors require further investigation.

Some important limitations of this meta-analysis should be considered when interpreting the results. First, the number of studies examined was small, and five of the studies were performed in the same institute, perhaps resulting in some duplication of cases. In addition, although all control groups were composed of cancer-free patients, inclusion criteria and baseline data for these groups varied from study to study. Second, any relevant articles that have not yet been published online might have been missed. Finally, due to significant heterogeneity, we could only roughly estimate the value of miR-210 in lung cancer diagnosis in this meta-analysis. In order to fully understand its potential clinical value, more studies are needed to further assess the diagnostic accuracy of miR-210 in different specimens, types, and stages of lung cancer.

In conclusion, we found that miR-210 had a moderate diagnostic value for lung cancer. Although miR- 210 expression alone may not be reliable enough for the clinical detection of lung cancer, panels that incorporate miR-210 along with other miRNAs or biomarkers could improve diagnostic efficiency.

## MATERIALS AND METHODS

### Search strategy

We systematically searched for articles published prior to April 20, 2016 in the Pubmed, Embase, Web of Knowledge, Cochrane Library, Chinese National Knowledge Infrastructure (CNKI), Chongqing VIP Information, and Wan Fang databases. Search terms were as follows: (“microRNA-210” or “miRNA-210” or “miR-210” or “miR210” or “hsa-mir-210”) and (“lung” or “pulmonary”) and (“neoplasm” or “neoplasms” or “neoplasia” or “cancer” or “cancers” or “carcinoma” or “tumor”). After relevant articles were identified, we examined their cited references to select other relevant articles. Articles were selected without regard to language. Two reviewers (Huqin Yang, Huijuan Wang) independently searched and evaluated the quality of the articles. Any disagreements between the two reviewers were resolved by a third person.

### Study selection

Inclusion criteria were as follows: 1) patients with any type of lung cancer; 2) inclusion of data on the diagnostic accuracy of miR-210 for lung cancer; 3) 2 × 2 contingency tables that could be directly extracted or calculated from the articles.

Exclusion criteria were as follows: 1, reviews, meta-analyses, letters, or expert opinions; 2, not related to miR- 210 or lung cancer; 3, not related to diagnostic value; 4) insufficient data.

### Data extraction and quality assessment

The following data were extracted from the eligible studies: first author, publication year, country, lung cancer type, stage, sample size, mean age, gender ratio, detection method, normalizer, specimen, sensitivity, specificity, true positive (TP), false negative (FP), false negative (FN), and true negative (TN). If any of these data were not mentioned in the articles, we obtained the missing information by contacting the corresponding authors. Study quality was evaluated according to QUADAS-2 guidelines [32].

### Statistical analysis

Statistical analyses were conducted using Stata (Stata Corporation, College Station, TX, USA, version 12.0) and Meta-disc (version 1.4) software. A bivariate random effects regression model was applied to calculate the pooled sensitivity, specificity, DOR, PLR, and NLR [33]. We also established a SROC curve and calculated AUCs and 95% confidence intervals [34]. An HSROC model was then used to confirm those data. Heterogeneity inspection was conducted using Cochran's *Q* test and Higgin's I-squared statistic [35]; an I^2^ greater than 50% indicated obvious heterogeneity between the studies. To explore heterogeneity, Spearman correlation coefficients and SROC analyses were conducted to determine whether there was a threshold effect. Meta-regression and subgroup analysis were used to explore sources of heterogeneity. Fagan's nomogram was employed to identify relationships between prior-test probability, likelihood ratio, and *post*-test probability [36]. Deeks' funnel plot asymmetry test was used to assess publication bias. In the linear regression test, a *p* value less than 0.1 indicated potential publication bias in our study [37].
